# *Moringa oleifera* leaf ethanolic extract benefits cashmere goat semen quality via improving rumen microbiota and metabolome

**DOI:** 10.3389/fvets.2023.1049093

**Published:** 2023-01-27

**Authors:** Jianyong Liang, Tiecheng Wu, Tao Wang, Yuejun Ma, Yurong Li, Shengguo Zhao, Yanli Guo, Bin Liu

**Affiliations:** ^1^College of Animal Science and Technology, Gansu Agricultural University, Lanzhou, China; ^2^Inner Mongolia Academy of Agricultural and Animal Husbandry Sciences, Hohhot, China; ^3^Engineering Laboratory of Genetic Resources Evaluation and Breeding Technology of Mutton Sheep in Inner Mongolia Autonomous Region, Hohhot, China; ^4^Alxa White Cashmere Goat Breeding Farm, Alxa League, China

**Keywords:** semen quality, cashmere goat, *Moringa oleifera* leaf extract, antioxidant, polyunsaturated fatty acid, microbiome, metabolome

## Abstract

**Background:**

Artificial insemination (AI) is an effective reproductive technique to improve the performance of cashmere goats and prevent the spread of diseases, and the quality of the semen determines the success of AI. The potential of *Moringa oleifera* leaf powder (MOLP) and *Moringa oleifera* leaf ethanolic extract (MOLE) to improve semen quality has been reported, but the underlying mechanisms remain unclear. For the purpose, 18 mature male cashmere goats were randomly assigned into three groups: the control (CON), MOLP, and MOLE groups. The CON group received distilled water orally; the MOLP group was orally treated with 200 mg/kg body weight (BW) MOLP; and the MOLE group was orally treated with 40 mg/kg BW MOLE.

**Results:**

Results showed that MOLE contained long-chain fatty acids and flavonoids. Treatment with MOLP and MOLE increased the activities of the serum catalase, superoxide dismutase, and glutathione peroxidase (*P* < 0.05), enhanced the total antioxidant capacity (*P* < 0.05), and reduced the serum malondialdehyde level (*P* < 0.05). At the same time, MOLE increased the contents of serum gonadotropin releasing hormone and testosterone (*P* < 0.05). Moreover, MOLE significantly increased sperm concentration, motility, and viability (*P* < 0.05). Meanwhile, MOLE raised the Chao1 index (*P* < 0.05) and altered the composition of the rumen microbiota; it also raised the relative abundance of *Treponema* (*P* < 0.05) and *Fibrobacter* (*P* < 0.05) and reduced the relative abundance of *Prevotella* (*P* < 0.1). Correlation analysis revealed the genus *Prevotella* was significantly negatively correlated with sperm concentration, as well as sperm motility and viability. Furthermore, MOLE significantly increased the rumen levels of the steroid hormones testosterone and dehydroepiandrosterone (*P* < 0.05), as well as the polyunsaturated fatty acids (PUFAs) alpha-Linolenic acid, gamma-Linolenic acid, docosapentaenoic acid, and 9-S-Hydroperoxylinoleicacid (*P* < 0.05).

**Conclusions:**

Oral MOLE supplementation can improve semen quality by increasing the antioxidant capacity and altering the rumen microbiota and metabolites of cashmere goats. Moreover, the MOLP supplementation could enhance the antioxidant capacity of cashmere goats.

## Graphical Abstract

MOLE supplementation can improve semen quality by increasing the antioxidant capacity and altering the rumen microbiota and metabolites of cashmere goats. 
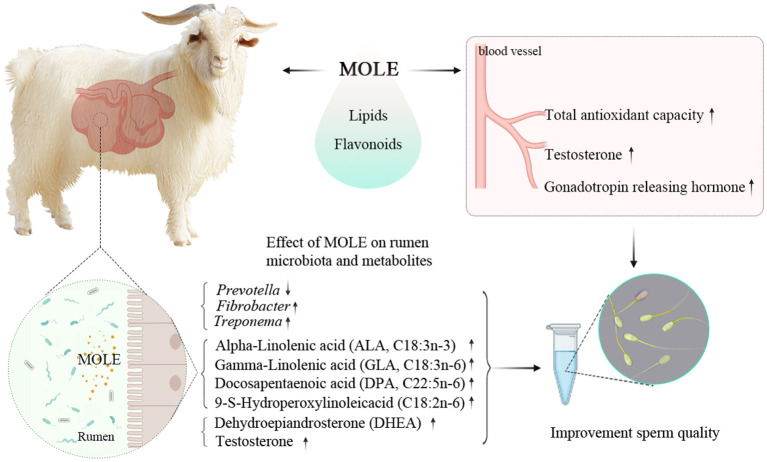

## Background

Cashmere goats are widely distributed in northern China, mainly providing meat and cashmere products for the local people. Cashmere is a unique and precious animal fiber that is of great importance in raising people's income (1). In recent years, owing to the degradation of natural populations as well as the rising cost of breeding stock supply and the growing demand for products (meat and cashmere), artificial insemination (AI) has become widely used in the cashmere goat industry. Semen quality is among the most critical elements for the AI's success. It can be influenced by multiple factors, including the age of the male and environmental factors (2). In addition, reactive oxygen species (ROS) are produced during the processing of semen, such as light (3) and temperature changes, which make sperm cells susceptible to oxidative stress (4, 5). The excessive production of reactive oxygen species causes different degrees of damage to sperm at the level of membrane, protein, and nucleic acid, which eventually leads to sperm death (6). Additionally, the plasma membrane of ruminant sperm contains a great number of PUFAs, and these PUFAs are susceptible to damage by oxidative stress, resulting in reduced semen quality (6). Increasing evidence shows that nutritional interventions could improve the semen quality of ruminants (7, 8). And also, the study by Shokry et al. showed *Moringa oleifera* leaf ethanolic extract (MOLE) supplementation at a level of 40 mg/kg body weight (BW) could effectively improve the oxidative status and semen quality of Barki rams (9).

*M. oleifera*, belonging to the monogeneric family Moringaceae (10), is a multipurpose plant and a comprehensive source of nutritional components like flavonoids and phenolic acids, PUFAs, tocopherols, minerals, and folate (11). *M. oleifera* leaves are enriched with a number of essential nutrients and other bioactive components, including potassium, calcium, phosphorous, iron, as well as known antioxidants such as polyphenols, ascorbic acid, and flavonoids (12). Moreover, studies have shown that *M. oleifera* leaves also contain omega-3 and omega-6 PUFAs, which are components of the sperm cell membrane, such as linoleic and linolenic acids (11, 13). The supplementation of *M. oleifera* leaves has been reported to improve sperm motility, plasma testosterone levels, and enhance libido in bulls (7). In addition, *M. oleifera* leaf extract can be utilized as a cryoprotectant for the cryopreservation of banana shrimp (14) and water buffalo sperm (15). It has also been used as a dietary supplement to improve the libido and sperm quality of rabbits (16). Recently, Zhang et al. showed that improving gut microbiota can affect sperm quality by regulating the function of the small intestine and the metabolome of plasma (17). Several other studies have also confirmed a causal relationship between the microbiota and spermatogenesis (18–21). However, whether *Moringa oleifera* leaf powder (MOLP) and MOLE can contribute to the semen quality of cashmere goats by modulating rumen microbiota and metabolites remains unclear. Therefore, we further estimated the rumen microbial composition, functions, and metabolites by using microbiome and metabolome analysis.

This study aimed to investigate the effects of oral MOLP and MOLE on the cashmere goat's semen quality and the underlying mechanisms, as well as the relationship between semen quality and rumen microbiota with metabolites.

## Materials and methods

### Cashmere goats, diets, and experimental design

**Figure 1:**
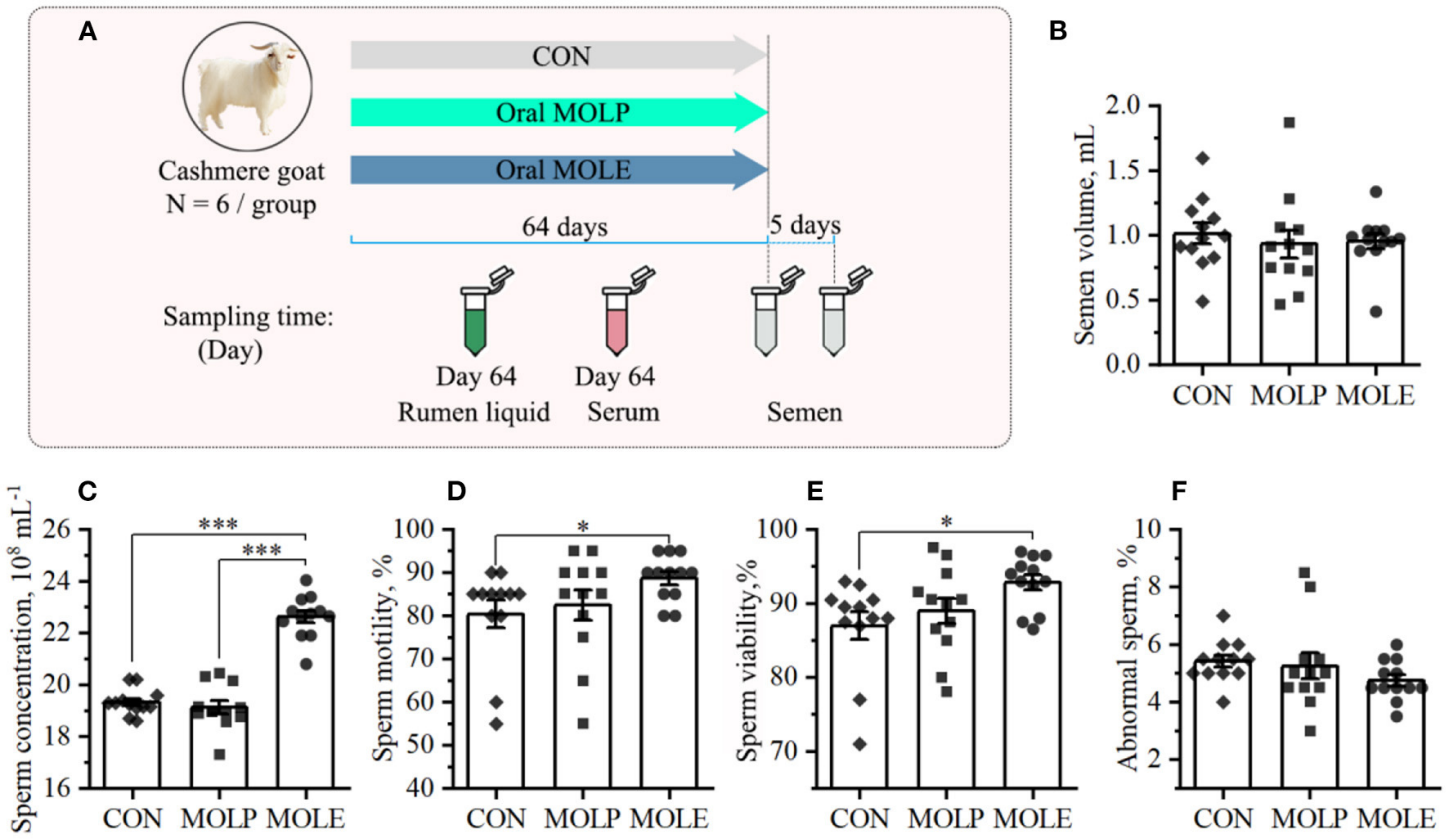
Effects of MOLP and MOLE on semen quality. **(A)** Schematic of study design. **(B)** Semen volume. **(C)** Sperm concentration. **(D)** Sperm motility. **(E)** Sperm viability. **(F)** Abnormal sperm. Data were expressed as the mean ± SEM. *P < 0.05; ***P < 0.001. CON, Control; MOLP, *Moringa oleifera* leaf powder; MOLE, *Moringa oleifera* leaf ethanolic extract.

The experiment was conducted at the Alxa White Cashmere Goat Breeding Farm (40°30′N, 105°30′E), Alxa League, China. A total of 18 male cashmere goats, aged 2-year-old and having an average BW of 46.3 ± 2.69 kg, were selected in this study. All the cashmere goats were placed in the same pen. Feeding goats with pellet feed provided daily nutritional demands according to the feeding standard [China, NY/T816, 2004 (22); Supplementary Table 1]. Clean tap water was offered *ad libitum*. Cashmere goats were randomly assigned into three groups of six goats each: the control (CON), MOLP, and MOLE groups. The CON group received distilled water orally; the MOLP group was orally treated with 200 mg/kg BW MOLP; and the MOLE group was orally treated with 40 mg/kg BW MOLE. Treatments were applied once daily in the afternoon for 64 days (Figure 1A). The applied MOLE dose was based on the previous work by Shokry et al. (9).

### Plant extraction and identification of chemical constituents

The nutritional ingredients of the MOLP obtained from commercial sources (Yunnan, China) are provided (Supplementary Table 2). MOLE was obtained from the method of El-Desoky et al. (16) with slight modification. In short, at room temperature, every 100 g of MOLP was extracted in 400 mL of 70% ethanol solution for at least 48 h. The extract was filtered through Whatman No. 1 filter paper. The collected filtrate was evaporated at 45°C until completely dry and then stored at −20°C until use.

The chemical composition of MOLE was analyzed by the UPLC-ESI (electrospray ionization)-MS/MS system of Shanghai Lu-Ming Biotechnology Co., Ltd. The MS operated in ESI positive ion and ESI negative ion modes. The ions were detected in full scan mode with a mass range of 100–1,200 m/z. Baseline filtering, peak identification, integration, retention time correction, peak alignment, and normalization were performed on the raw LC-MS data with the software Genesis QI V2.3 (Nonlinear, Dynamics, UK). The obtained mass-to-charge ratios (m/z), secondary fragments, and isotopic distributions were used to identify the species of compounds, which were then qualitatively analyzed using Lipid Maps (Version 2.3), the Human Metabolome Database (HMDB), METLIN, and self-built databases.

### Semen collection and analysis

Semen samples were collected twice from each goat on days 64 and 69 by a pre-warmed artificial vagina, and almost all samples were processed within 45 min of collection. Semen parameters were analyzed according to the method described by El-Desoky et al. (16). Briefly, the volume of cashmere goat semen was calculated by weighing (assuming a semen density of 1 g/mL). Sperm concentration was assessed with the modified Neubauer hematocrit plate. The percentage of live, dead and abnormal spermatozoa was assessed by counting 200 sperm cells with the eosin-aniline black staining method. Completely or partially purple stained sperm cells were considered dead, while unstained sperm cells were considered live. Sperm motility was performed in several microscopic fields for each semen sample by a visual examination under 200 × magnification using a light microscope with heated stage and subjectively assessed from 0 to 100%.

### Serum sampling and measurement

Blood serum samples were collected on day 64 with a 5 mL vacuum blood collection tube from the jugular vein of each goat. Blood samples were collected and placed at room temperature for 30 min, then centrifuged at 3,000 rpm for 10 min to separate the serum and stored at −20°C until analysis. The concentrations of serum gonadotropin-releasing hormone (GnRH) and testosterone were measured by enzyme-linked immunosorbent assay (ELISA) with the ELISA kit. We measured catalase (CAT) activity by the ammonium molybdate method, total superoxide dismutase (SOD) activity by the xanthine method, glutathione peroxidase (GSH-Px) activity by colorimetry, total antioxidant capacity (T-AOC) by the 2,2-azino-bis-3-ethylbenzothiazoline-6-sulfonic acid (ABTS) method, and malondialdehyde (MDA) content by the thiobarbituric acid (TBA) method. All kits were purchased from Beijing Huaying Institute of Biotechnology (China), and the operating procedures were performed according to the manufacturer's instructions.

### Rumen liquid sampling

On day 64, rumen liquid was sampled from each goat using an oral stomach tube connected to a vacuum pump before morning feeding. The tube was inserted into the central rumen to reduce contamination. The collected rumen samples were filtered through four layers of sterile gauze to obtain rumen liquid (23). Then, 10 mL of rumen fluid was immediately transferred to sterile lyophilization tubes, plunged into liquid nitrogen for freezing, and then transferred to −80°C to be stored for DNA extraction and metabolome analysis.

### Microbiome analysis

Total genomic DNA of rumen liquid from cashmere goats was extracted with the MagPure Soil DNA LQ Kit (Meigen Biotech, Guangzhou, China), following the instructions of the manufacturer. The DNA concentration was checked with a NanoDrop2000 (Thermo Fisher), and DNA quality was verified with a 1% agarose gel. For bacterial diversity analysis, PCR amplification of the V3-V4 variable regions of the 16S rRNA gene was performed using bacterial forward and reverse primers 343F (5′-TACGGRAGGCAGCAG-3′) and 798R (5′-AGGGTATCTAATCCT-3′), respectively (24). PCR was amplified in a 30 μL reaction mixture, including PCR premix (15 μL), each primer (1 μL), DNA template (50 ng), and PCR grade-water to adjust the volume. After the PCR amplicon library was prepared, the library sequencing was conducted using the Illumina MiSeq sequencing platform by OE Biotechnology Co., Ltd. in Shanghai.

The raw data was generated using Illumina MiSeq sequencing, and Trimmomatic software (25) was used to cut off sliding windows with base mass averages <20 and to remove sequences <50 bp in length. Then use Flash software (26) to splice the qualified raw data from the previous step to obtain the complete paired-end sequence. Further, QIIME software (27) was used to remove sequences containing bases (N) from the paired end sequences, to remove sequences with single base repeats >8, and to remove sequences <200 bp in length to obtain clean tag sequence. Finally, we use the UCHIME software (28) to remove chimeras fromclean tags and obtain valid tags for later OTU delineation. Detailed statistical information about the sequencing data is shown in Supplementary Table 3. The valid tags obtained from quality control were subjected to OTU classification with Vsearch software (29), and the representative sequences were annotated with the Silva database using the RDP classifier (30) to obtain the OTU annotation information.

### Metabolome analysis

Rumen liquid sample processing: 500 μL of rumen liquid was transferred into a 1.5 mL centrifuge tube and centrifuged (15 min, 13,000 rpm, 4°C), then 100 μL of supernatant was transferred into another 1.5 mL tube. Then, 20 μL of internal standard L-2-chlorophenylalanine-methanol (0.3:1, m:v) and 400 μL of methanol-acetonitrile (2:1, v:v) were added to the centrifuge tube. The mixture was processed by vortex shaking (1 min), sonication in an ice-water bath (10 min), standing (30 min, −20°C), and then centrifugation (10 min, 13,000 rpm, 4°C). The 200 μL supernatant was transferred into a brown LC-MS vial to volatilize and dry, then re-dissolved with 300 μL of methanol-water solution (1:4) (sonication for 3 min), followed by storage at −20°C for 2 h; after centrifugation (10 min, 13,000 rpm, 4°C), 150 μL of supernatant was transferred into a brown LC-MS vial and stored at −80°C.

The treated rumen liquid samples were analyzed by Lu-Ming Biotech Co., Ltd. (Shanghai, China). The liquid chromatography–mass spectrometry (LC–MS) system used in this experiment was composed of an AB ExionLC (AB Sciex) ultra-high performance liquid tandem with an AB TripleTOF 6600 plus (AB Sciex) high-resolution mass spectrometer. A chromatographic column, ACQUITY UPLC HSS T3 (Waters), was used, and the column temperature was set at 45°C. The mobile phase A was water (containing 0.1% formic acid) and phase B was acetonitrile (containing 0.1% formic acid) at a controlled flow rate of 0.35 mL/min. The mass spectrometry ion source was ESI, and the acquisition was performed in positive and negative ion scanmodes, respectively, with a mass scan range (product ion scan, m/z) of 40–1,000.

Baseline filtering, peak identification, integration, retention time correction, peak alignment, and normalization were performed on the raw LC-MS data with the software Genesis QI V2.3 (Nonlinear, Dynamics, UK). The obtained mass-to-charge ratios (m/z), secondary fragments, and isotopic distributions were used to identify the species of compounds, which were then qualitatively analyzed using Lipid Maps (Version 2.3), the Human Metabolome Database (HMDB), METLIN, and self-built databases. Metabolites were analyzed and compared between the two groups using the Student's *t*-test and fold change, and pathway enrichment analysis was performed using the online KEGG database (https://www.kegg.jp/).

### Statistical analysis

The statistical analysis software used in this study was SPSS (version 19.0). The results were expressed as mean ± SEM. Comparisons between two groups were made using the Student's *t*-test. Comparisons between three groups were made using a one-way ANOVA followed by *post-hoc* tests using LSD multiple comparisons. *P* < 0.05 were considered statistically significant, *P* < 0.01 and *P* < 0.001 were considered extremely statistically significant, and 0.05 < *P* < 0.10 was considered a trend toward a significant difference. The correlation analysis was performed using Spearman's correlation coefficient. Furthermore, Origin2021 (OriginLab, USA) was used for graphing.

## Results

### Bioactive constituents of MOLE

Altogether, a total of 17 secondary metabolites were preliminarily identified in MOLE through analytical characterization. Flavonoids, fatty acyls compounds, and glycerophospholipids account for the main components of the extract, including catechin, rutin, quercetin, isoquercitrin, kaempferol 3-O-rutinoside, and quercetin 3-arabinoside (Table 1).

**Table 1:** Chemical composition of MOLE detected by LC-MS.

**Retention time, min**	**M+H^+^(m/z)**	**MS/MS**	**Tentatively identified compounds**	**Chemical formula**	**Molecular weight**	**Class**
4.50	291	123,139,165,291	Catechin	C_15_H_14_O_6_	290	Flavonoids
5.05	611	465,449,303,611	Rutin (Quercetin 3-O-rutinoside)	C_27_H_30_O_16_	610	
5.07	303	137,153,229,303	Quercetin	C_15_H_10_O_7_	302	
5.19	465	85,257,303,465	Isoquercitrin	C_21_H_20_O_12_	464	
5.26	595	85,287,449,595	Kaempferol 3-O-rutinoside	C_27_H_30_O_15_	594	
5.34	435	73,303,435	Quercetin 3-arabinoside	C_20_H_18_O_11_	434	
6.45	303	137,153,229,303	Quercetin	C_15_H_10_O_7_	302	
10.34	222	81,121,222	N-Isobutyl-2,4,8-decatrienamide	C_14_H_23_NO	221	Fatty acyls
12.81	256	57,88,181,256	Palmitic amide	C_16_H_33_NO	255	
13.85	334	57,334	2,4,12-Octadecatrienoic acid isobutylamide	C_22_H_39_NO	333	
14.87	362	57,289,362	2,4,14-Eicosatrienoic acid isobutylamide	C_24_H_43_NO	361	
11.08	496	104,184,496	LysoPC (0:0/16:0)	C_24_H_50_NO_7_	495	Glycerophospholipids
13.06	782	86,184,782	PC [20:4(5Z,8Z,11Z,14Z)/16:0]	C _44_ H _80_ NO _8_ P	781	
13.69	782	86,184,782	PC [16:0/20:4(5Z,8Z,11Z,14Z)]	C _44_ H _80_ NO _8_ P	781	
9.82	286	135,201,286	Piperine	C_17_H_19_NO_3_	285	Alkaloids
11.24	342	86,135,229,342	Pipernonaline	C_21_H_27_NO_3_	341	Benzodioxoles
14.64	346	69,86,112,261,346	2,4,12-Octadecatrienoic acid piperidide	C_23_H_39_NO	345	Piperidines

### Effects on semen quality

The primary indicators of semen quality (semen volume, sperm concentration, motility, viability, and abnormality rates) were measured in this study. Figure 1 shows the semen parameters and characteristics of the three treatment groups. Compared with the CON group, MOLE supplementation significantly increased sperm concentration, motility, and viability (Figures 1C–E), but did not change semen volume or the percentage of abnormal sperm (Figures 1B, F).

### Effects on blood serum biochemical properties

**Figure 2:**
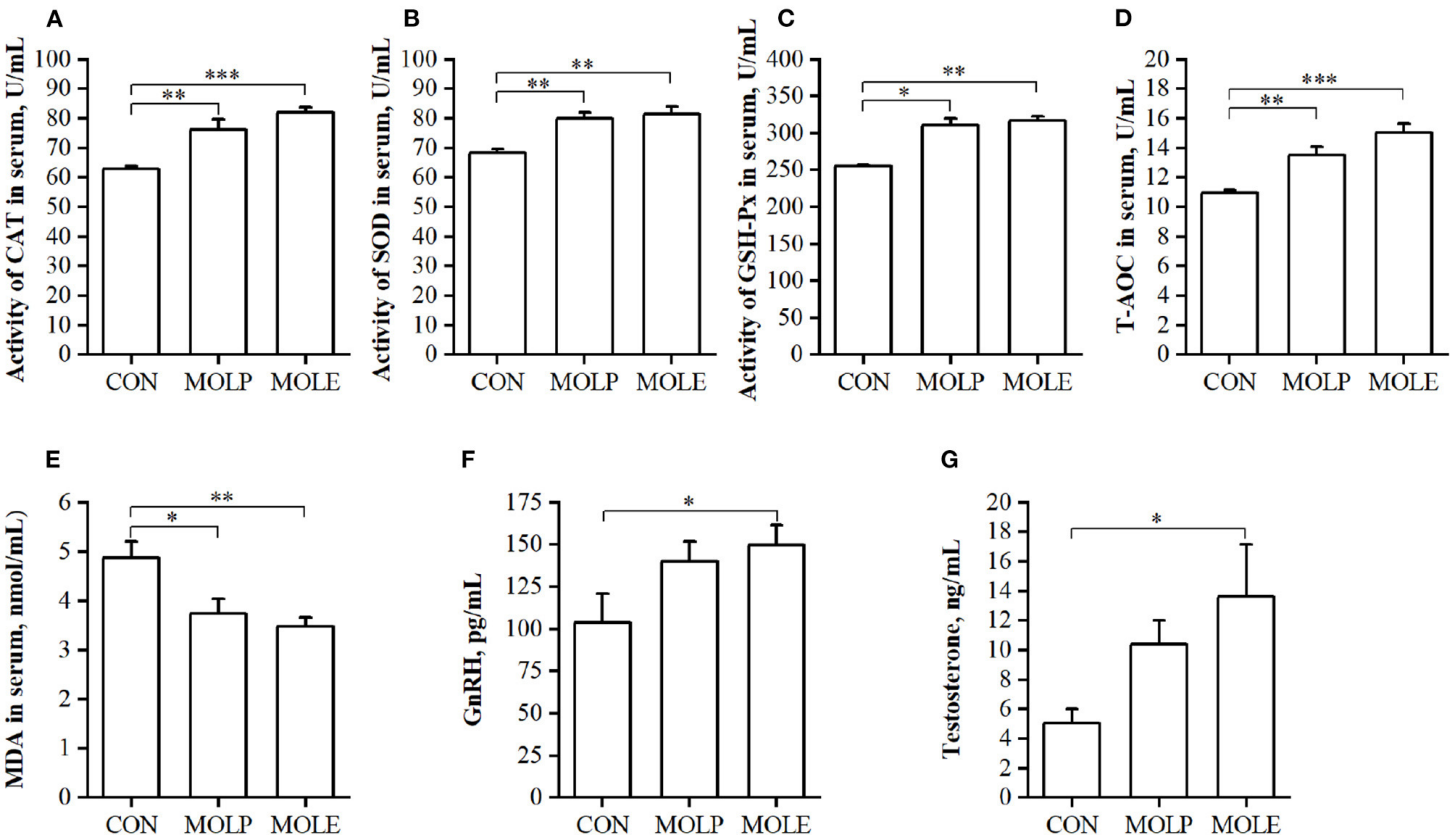
Effect of MOLP and MOLE supplementation on serum antioxidant capacity and reproductive hormone of cashmere goats. **(A)** CAT; **(B)** SOD; **(C)** GSH-Px; **(D)** T-AOC; **(E)** MDA; **(F)** GnRH; and **(G)** Testosterone. The values in the histogram are the means ± SEM, *P < 0.05; **P < 0.01; ***P < 0.001. CON, Control; MOLE, *Moringa oleifera* leaf ethanolic extract; MOLP, *Moringa oleifera* leaf powder; CAT, Catalase; SOD, Superoxide dismutase; GSH-Px, Glutathione peroxidase; T-AOC, Total antioxidant capacity; MDA, Malondialdehyde; GnRH, Gonadotropin releasing hormone.

The effects of MOLP and MOLE supplementation on serum antioxidant capacity and reproductive hormones in cashmere goats are shown in Figure 2. MOLE and MOLP supplementation significantly increased CAT, SOD, and GSH-Px activities and T-AOC in the serum of cashmere goats (Figures 2A–D, *P* < 0.05), whereas MDA levels in the serum were significantly decreased (Figure 2E, *P* < 0.05). In addition, MOLE was found to have an effect on the serum levels of reproductive hormones in cashmere goats. The results showed that serum GnRH and testosterone levels were significantly higher in the MOLE group than in the CON group (Figures 2F, G, *P* < 0.05).

### Effects on diversity and composition of rumen microbes

The metadata of the 16S rRNA gene sequencing of rumen liquid DNA was summarized in Supplementary Table 3. After quality control of the raw data obtained from sequencing, a total of 1,319,997 pairs of clean tags were obtained, and 1,195,946 valid tags (with an average length of 419 bp) were obtained after removing chimeras from clean tags. There were 8,233 common OTUs in the CON, MOLP, and MOLE groups. Moreover, the CON, MOLP, and MOLE groups contained 852,738 and 787 unique OTUs, respectively (Supplementary Figure 1D).

We analyzed bacterial 16S rDNA sequences in rumen liquid of cashmere goats and showed that the rarefaction curve variation tended to be horizontal, indicating that the samples were reasonably sequenced for further analysis (Supplementary Figure 1A). Therefore, the level of microbial diversity within (α-diversity) and between (β-diversity) groups of different treatments was analyzed. As for α-diversity, we applied the Chao1 and Shannon diversity indices (Supplementary Figures 1B, C). The study found that the Chao1 index (*P* < 0.05) and Shannon index (*P* > 0.05) were higher in the MOLE group than those in the CON group, indicating that MOLE increased the α-diversity of rumen microbes in cashmere goats. Regarding β-diversity, significant clustering was observed in the composition of the microbiota of the CON and MOLE groups using binary-Jaccard-based principal coordinate analysis (PCoA) (*P* < 0.05, Supplementary Figure 1E).

**Figure 3:**
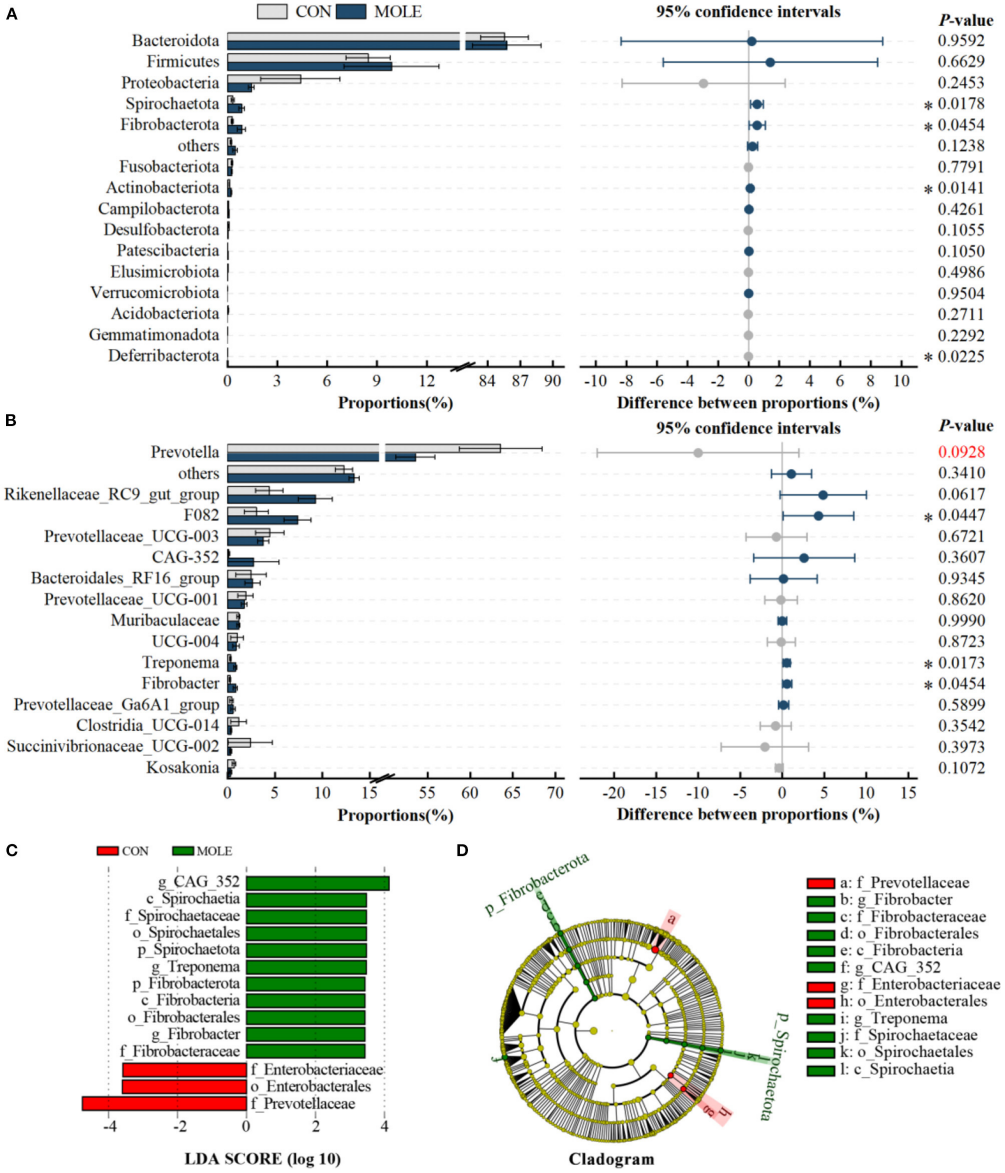
Effects of MOLE on the rumen microbial composition. The difference between bacterial phyla **(A)** and genera **(B)** was tested by the Student's *t*-test. The top 15 microbes' average abundance was compared. **(C)** Linear Discriminant Analysis (LDA) coupled with effect size (LEfSe) measurements identifies the most differentially abundant taxa between CON and MOLE groups. Taxa enriched in the MOLE group are indicated with a positive LDA score (green), and taxa enriched in the CON group have a negative score (red). Only taxa with an LDA score of > 3 and a *P* value of < 0.05 are shown. **(D)** Cladogram for rumen microbiota (CON group vs. MOLE group). The values in the histogram are the mean values of relative abundance ± SEM. Trend for 0.05 < *P* < 0.1, and *P < 0.05.

In addition, we analyzed differences in the rumen microbiome at the phylum and genus levels. At the phylum level, the relative abundance of *Spirochaetota, Fibrobacterota*, and *Actinobacteriota* was significantly higher in the MOLE group than in the CON group, while that of *Deferribacterota* was lower (*P* < 0.05, Figure 3A). At the genus level, the abundance of *F082, Treponema*, and *Fibrobacter* were significantly higher in the MOLE group than in the CON group (*P* < 0.05), while that of *Prevotella* tended to be lower (53.59 vs. 63.61, *P* < 0.1, Figure 3B). In addition, linear discriminant analysis (LDA) coupled with effect size (LEfSe) measurements further identified rumen microbial taxa at the phylum to genus level. The LEfSe analysis showed a significant difference in rumen microbial composition between the MOLE and CON groups (LDA > 3, *P* < 0.05). Higher abundances of *Spirochaetaceae* and *Fibrobacteraceae* were found in the MOLE group, while *Prevotellaceae* and *Enterobacteriaceae* were more abundant in the CON group; The abundances of *CAG_352, Treponema*, and *Fibrobacter* were higher in the MOLE group, while *Enterobacterales* were more abundant in the CON group (Figures 3C, D).

**Figure 4:**
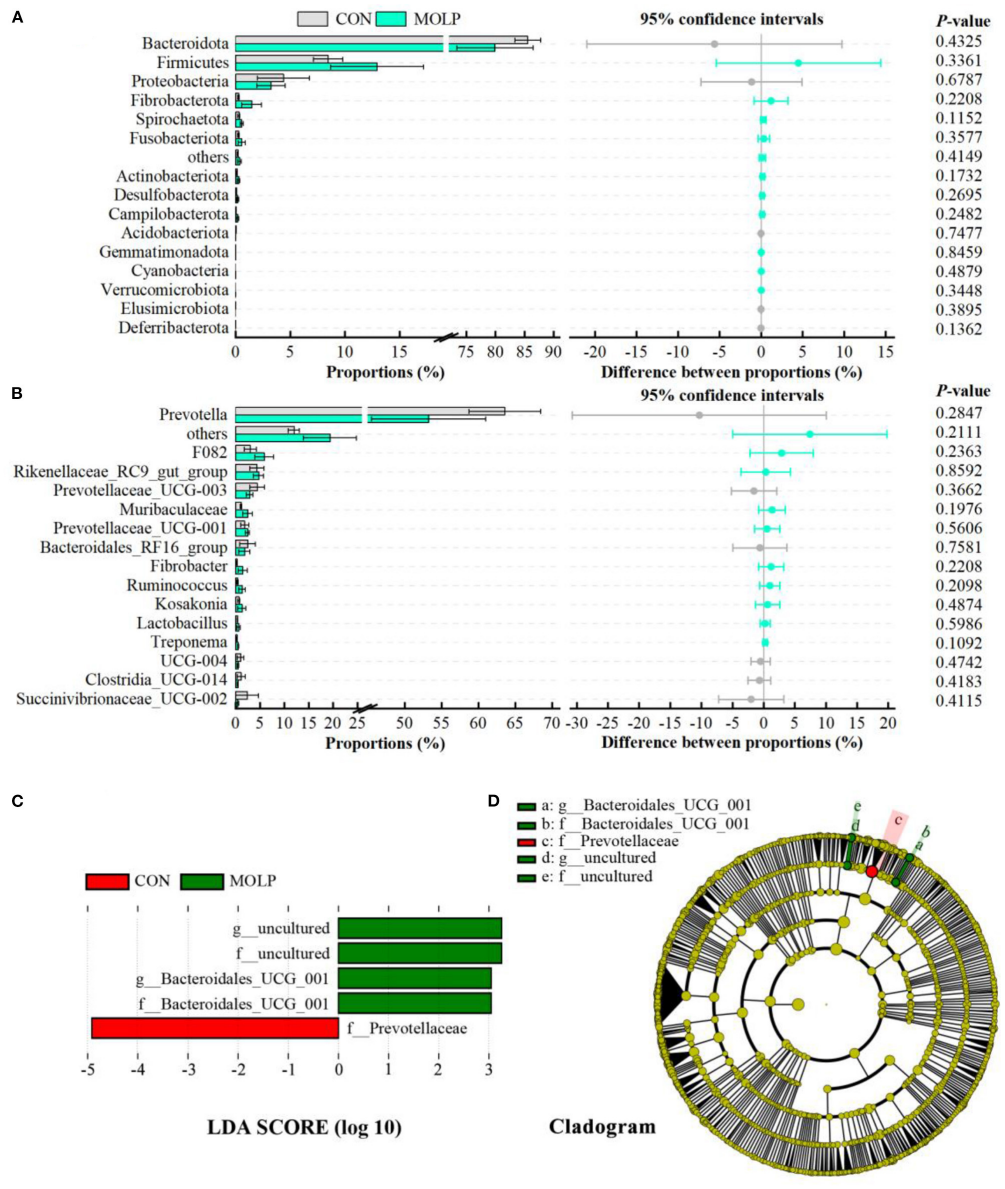
Effects of MOLP on the rumen microbial composition. The difference between bacterial phyla **(A)** and genera **(B)** was tested by the Student's *t*-test. The top 15 microbes' average abundance was compared. The values in the histogram are the mean values of relative abundance ± SEM. **(C)** Linear Discriminant Analysis (LDA) coupled with effect size (LEfSe) measurements identifies the most differentially abundant taxa between CON and MOLP groups. Taxa enriched in the MOLP group are indicated with a positive LDA score (green), and taxa enriched in the CON group have a negative score (red). Only taxa with an LDA score of > 3 and a *P* value of < 0.05 are shown. **(D)** Cladogram for rumen microbiota (CON group vs. MOLP group).

However, there were no differences in the MOLP and CON groups at the bacterial phylum and genus levels (Figures 4A, B). Notably, in the MOLP group, the *Prevotella* genus showed lower abundances than in the CON group (53.27 vs. 63.61, *P* > 0.1, Figure 4B). Furthermore, the LEfSe measurements further identified rumen microbial taxa at the phylum to genus levels (LDA > 3, *P* < 0.05). Figures 4C, D show that the abundance of *Bacteroidales_UCG_001* was higher in the MOLP group, while that of *Prevotellaceae* was higher in the CON group.

### Effects on the rumen metabolites

**Figure 5:**
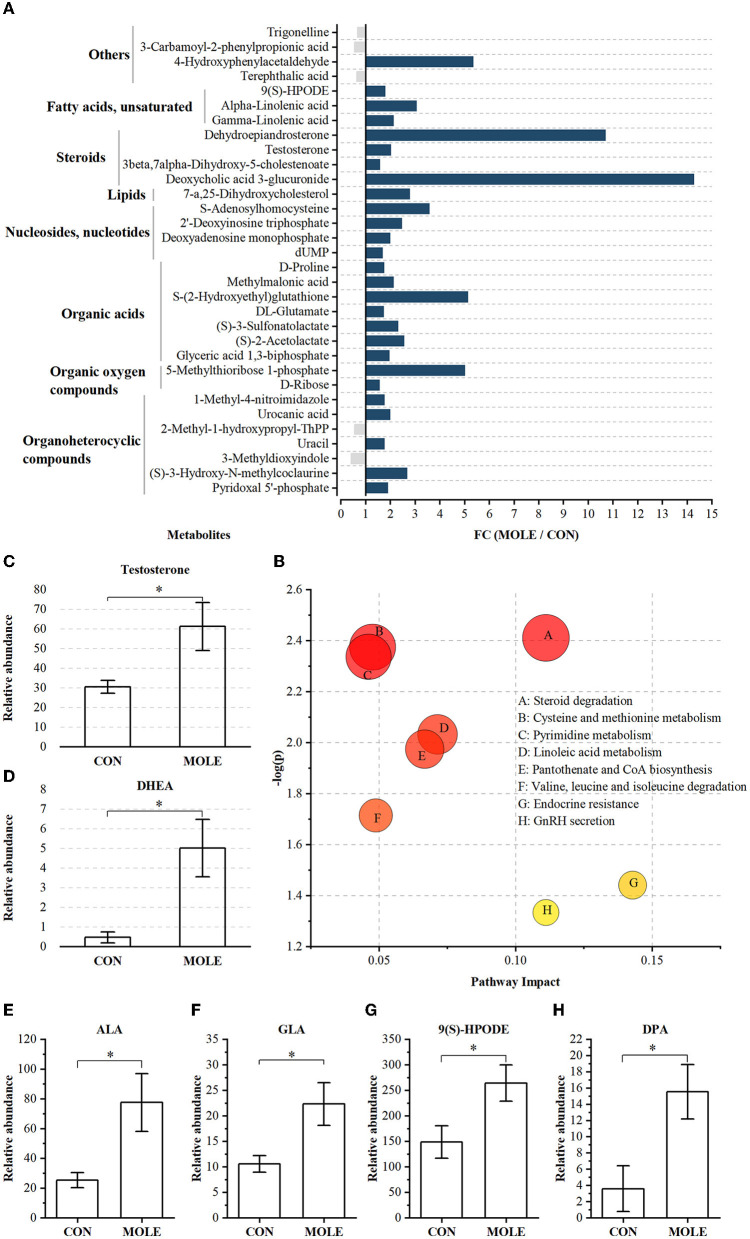
The alteration of rumen metabolites between MOLE and CON groups. **(A)** MOLE/Control fold change (FC) of rumen metabolites with significant differences between MOLE and CON groups (*P* < 0.05 and FC > 1.5 or FC < 1/1.5). **(B)** Pathway enrichment analysis was performed using the significantly different rumen metabolites between the MOLE and CON groups. Relative abundance of **(C)** testosterone, **(D)** DHEA, **(E)** ALA, **(F)** GLA, **(G)** 9(S)-HPODE, and **(H)** DPA. The values in the histogram are the means ± SEM. *P < 0.05. ALA, Alpha-Linolenic acid; GLA, Gamma-Linolenic acid; DPA, Docosapentaenoic acid; 9(S)-HPODE, 9-S-Hydroperoxylinoleicacid; DHEA, dehydroepiandrosterone.

A total of 1,006 compounds were identified in the rumen metabolome (Supplementary Table 4). After the combined analysis of Student's *t*-test and fold change (FC), 44 metabolites were significantly increased (*P* < 0.05 and FC > 1.5) and 11 metabolites were significantly reduced (*P* < 0.05 and FC < 1/1.5) in the rumen of cashmere goats supplemented with MOLE, compared with the CON group (Supplementary Table 5). Using KEGG analysis, 32 differential metabolites were enriched in metabolic pathways (Figure 5A), including steroid degradation, cysteine and methionine metabolism, pyrimidine metabolism, linoleic acid metabolism, pantothenate and CoA biosynthesis, valine, leucine and isoleucine degradation, endocrine resistance, and GnRH secretion (*P* < 0.05, Figure 5B). Notably, MOLE increased testosterone and dehydroepiandrosterone (DHEA) levels in the rumen liquid of cashmere goats (*P* < 0.05, Figures 5C, D). Furthermore, it increased the relative abundance of PUFAs such as Alpha-Linolenic acid (ALA, C18:3n-3), Gamma-Linolenic acid (GLA, C18:3n-6), Docosapentaenoic acid (DPA, C22:5n-6), and 9-S-Hydroperoxylinoleicacid (9(S)-HPODE, C18:2n-6) (*P* < 0.05, Figures 5E–H).

Similarly, comparison analysis after the Student's *t*-test and FC indicated that the relative abundances of 31 metabolites were increased in the rumen of MOLP cashmere goats (*P* < 0.05 and FC > 1.5, Supplementary Table 6), and that of 24 metabolites were reduced (*P* < 0.05 and FC < 1/1.5). Pathway enrichment analysis using KEGG IDs for 55 differential metabolites yielded five metabolic pathways, including galactose metabolism, ABC transporter, valine, leucine, and isoleucine biosynthesis, linoleic acid metabolism, and PPAR signaling pathway (*P* < 0.05; Supplementary Figure 2).

### Correlation of rumen microbiome, rumen metabolome, and semen quality

**Figure 6:**
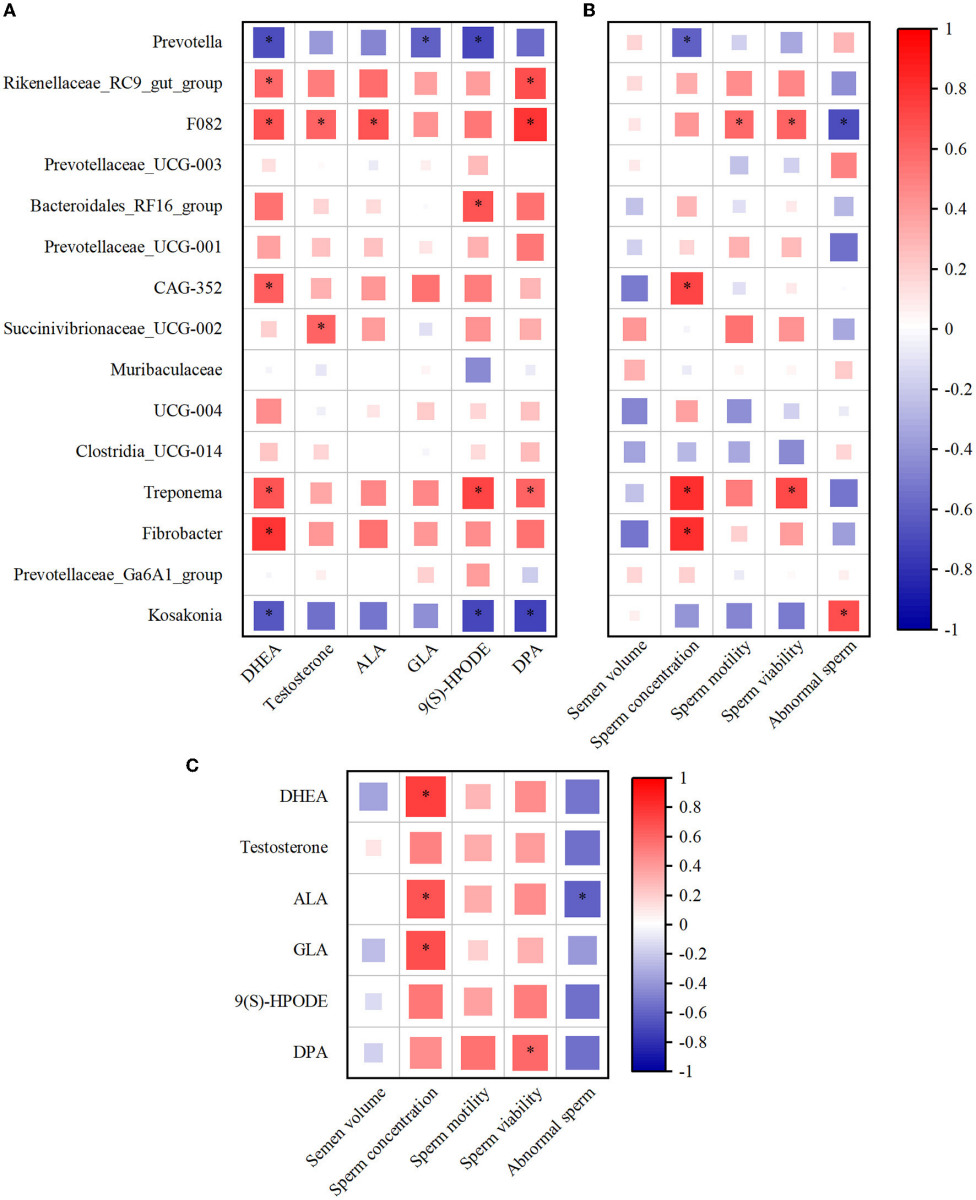
The alteration of rumen microbiota and metabolites was closely associated with the semen quality. **(A)** Spearman's correlation analysis of top15 genus microbiota levels with DHEA, testosterone, ALA, GLA, 9(S)-HPODE, and DPA levels. **(B)** Spearman's correlation analysis of top15 genus microbiota levels with semen quality parameters (the average of semen quality of each goat obtained from the last two collections before the end of the experiment). **(C)** Spearman's correlation analysis of semen quality parameters with DHEA, testosterone, ALA, GLA, 9(S)-HPODE, and DPA levels. Red indicates a positive correlation, and blue indicates a negative correlation. *P < 0.05. DHEA, dehydroepiandrosterone; ALA, Alpha-Linolenic acid; GLA, Gamma-Linolenic acid; DPA, Docosapentaenoic acid; 9(S)-HPODE, 9-S-Hydroperoxylinoleicacid.

The Spearman's correlation coefficient was used to study the functional relationship among rumen microbiota, metabolites, and semen quality. As illustrated in Figure 6A, Spearman's correlation analysis of top15 genus-level rumen bacteria with DHEA, testosterone, ALA, GLA, 9(S)-HPODE, and DPA revealed that *Prevotella* was negatively correlated with DHEA, GLA, and 9(S)-HPODE (*P* < 0.05), while testosterone, ALA, and DPA were negatively correlated but not significantly with rumen *Prevotella*. In contrast, the *Treponema* positively correlated with DHEA, 9(S)-HPODE, and DPA (*P* < 0.05), and *Fibrobacter* positively correlated with DHEA (*P* < 0.05, Figure 6A). In addition, *Prevotella* was significantly negatively correlated with sperm concentration (*P* < 0.05), and negatively correlated with sperm motility and viability but not significant (Figure 6B). Conversely, *Treponema* was positively correlated with sperm concentration and viability (*P* < 0.05), and *Fibrobacter* was positively correlated with sperm concentration (*P* < 0.05, Figure 6B). When combining rumen metabolites and semen quality, the analysis showed a significantly positive correlation between the sperm concentration and the rumen DHEA, ALA, and GLA (*P* < 0.05). In addition, there was a positive correlation between sperm viability and DPA and a negative correlation between abnormal sperm and ALA (*P* < 0.05, Figure 6C).

## Discussion

Nutrients-rich *M. oleifera* and its leaves are commonly used for animal nutrition or medicinal purposes (31). Its leaves are rich in minerals, proteins, vitamins, and antioxidants (32), as well as polyphenols such as quercetin, isoquercitrin (33), rutin (34), kaempferol (35), and other polyphenols (36). According to several prior studies, MOLP and MOLE contain several plant-based bioactive compounds with antibacterial, antioxidant, and anti-immune activities that improve reproductive performance and health in animals (7, 37–39). However, the specific mechanism of these effects remains unclear. Therefore, we further evaluated the rumen microbial composition, functions, and metabolites using the rumen microbiome combined with the metabolome.

In this study, analysis of MOLE chemical constituents showed that MOLE major contained flavonoids, fatty acyls, and glycerophospholipids. It is interesting to note that MOLE contained rutin, a well-known antioxidant that has superoxide radical scavenging ability (40). Moreover, other phytogenic compounds with different antioxidant activities, such as quercetin, isoquercitrin, kaempferol 3-O-rutinoside, quercetin 3-arabinoside, and catechin were also detected. Therefore, this coincided with an increase in serum antioxidant enzyme (CAT, SOD, and GSH-Px) activity and T-AOC and a reduction in serum MDA concentrations. Similarly, a study by El-Desoky et al. showed MOLE supplementation improved the antioxidant capacity of rabbits (16). Moreover, recent research revealed that the use of nanoencapsulated MOLE increased the T-AOC of rabbits (39). In this study, we found that MOLP supplementation also increased serum antioxidant levels in cashmere goats, similar to the previously reported results (41). Overall, supplementation with MOLP and MOLE may strengthen the antioxidant defense system of cashmere goats by scavenging free radicals produced from oxidation processes.

We further investigated the effect of oral supplementation with MOLP and MOLE on semen quality in cashmere goats and showed that MOLE significantly increased sperm concentration but had no effect on semen volume. This is attributed to enhanced testosterone synthesis, as testosterone is an androgen necessary to promote spermatogenesis (42). Our study also found that oral supplementation with MOLE improved sperm quality, such as sperm motility and viability. These results are consistent with previous research findings that MOLE increases sperm quality in rats (38) and rams (9). The improvement of sperm quality may be related to the fact that the antioxidant bioactive components in MOLE enhanced the antioxidant status of cashmere goats, mitigating the harmful effects of reactive oxygen species. Moreover, MOLE supplementation significantly increased the content of PUFAs in the rumen, which is an important component of the plasma membrane of sperm cells, with beneficial effects on the improvement of sperm motility and viability (43). As previously reported, supplementation of the diet with PUFAs, especially ω-3 PUFAs, is beneficial in improving semen quality in rats (44), birds (45–47), bulls (8), and boars (48, 49). The results of the correlation analysis support this argument.

Androgens (mainly testosterone) are synthesized mainly in the Leydig cells of the testis and are critical for spermatogenesis and the maintenance of reproductive performance in cashmere goats (50, 51). In the current study, we found that MOLE supplementation increased serum testosterone concentrations significantly in cashmere goats. However, the effect of MOLP on serum testosterone in cashmere goats shows the same trend, but the effect was not significant. Previous studies have shown that oral administration of *M. oleifera* leaf extract increases testosterone levels in rats (38). Besides, *in vitro* studies have found that *M. oleifera* leaf extract promotes testosterone production in the Leydig cells (52). The effect of MOLE on testosterone is related to the presence of flavonoids in MOLE that have been shown to alter androgen levels (53), such as quercetin which has been studied to promote androgen production in Leydig cells by modulating Star gene expression and Star promoter activity (54). In addition, testosterone levels have been reported to be closely related to the composition and diversity of gut microbes (55), with higher testosterone levels in animals with high gut microbial diversity (56). Given the scope of this study, our experimental results are similar to previous results in other animals. In short, MOLE supplementation led to an increase in the diversity of the rumen microbiome, which increasing the serum testosterone concentration in cashmere goats.

Recent studies have suggested that gut microbiota can have an impact on spermatogenesis (18–20). It is well known that MOLE and MOLP can enhance sperm quality and testosterone levels (7, 37, 52), but there is limited information about their effects on the rumen microbiota. In this study, we found that in the rumen treated with MOLE, the abundance of gram-negative bacteria, *Prevotella*, tended to decrease. After further analysis by LEfSe, we found that the relative abundance of *Prevotellaceae* was significantly reduced in the MOLE group. Correlation analysis showed that the *Prevotella* had a negative correlation with sperm concentration, viability, and motility. Similarly, a recent paper reported that hydroxytyrosol, a polyphenol, reduced the abundance of the gut Prevotellaceae and then improved spermatogenesis and sperm motility (19). And clinically, Ding et al. reported that *Prevotella* had a negative correlation with subjects' sperm motility, suggesting that *Prevotella* could play a key role in regulating sperm production (18). The results of the correlation analysis support this argument. Moreover, the relative abundance of *Fibrobacteriaceae* in the MOLE group has increased, which may be related to the increased serum testosterone level (57). Additionally, we detected a significant increase in the relative abundance of *Treponema* in the MOLE group. However, their complex mechanisms of action are still unclear and require further study.

The metabolic processes of rumen microbiota can provide vital nutrients to the reproductive system. In this study, MOLE supplementation altered the rumen metabolites such as PUFAs and steroid hormones in cashmere goats. Many studies have shown that PUFAs are a major component of sperm cell membrane lipids and play a crucial role in regulating sperm function by altering the integrity and fluidity of the sperm plasma membrane (43, 44, 58, 59). Notably, the rumen metabolomic analysis revealed that supplementation of MOLE significantly increased the relative abundance of PUFAs in cashmere goat's rumen, including ALA, GLA, DPA, and 9(S)-HPODE. In mammalian cells, ALA can be converted to docosahexaenoic acid (DHA, C22:6n-3) by alternating steps of longation and desaturation (43). DHA has been identified as an important component of ruminant sperm membrane phospholipids (60, 61), mainly distributed in the head and tail of sperm, while the tail is mainly associated with sperm motility and the head with acrosome reaction and membrane fusion (62–64). In summary, ALA improves sperm function in cashmere goats primarily by altering the fatty acid profile of the sperm head and tail. Moreover, in this study, we found that supplementation with MOLE enhanced the serum testosterone level and the content of serum GnRH. This result was also associated with alterations in PUFAs, which can have a direct effect on steroid acute regulators and cytochrome P450 (65). Also, 20-carbon PUFAs act as precursors for prostaglandin synthesis and are directly involved in the regulation of reproductive endocrinology (66–68). Thus, PUFAs play an important role in promoting testosterone biosynthesis. Interestingly, rumen metabolome analysis showed an increase in the relative abundance of steroid hormones and their derivatives, especially testosterone and DHEA, following MOLE supplementation. Androgen biosynthesis and metabolism have been extensively studied in the past, but the mechanism of androgen metabolism in the rumen of ruminants remains unclear. Traditionally, androgens are produced by the testes, metabolized by the liver, and subsequently excreted by the kidneys (69). However, Collden et al. showed that the gut microbiota can regulate androgen metabolism (70). Hepatic excretion of glucuronidated androgens can be efficiently deglucuronidated by the gut microbiota, resulting in the presence of most androgens in the gut in their prototype form (70, 71). This may explain the elevated rumen testosterone and DHEA levels after MOLE supplementation.

To our knowledge, this study is the first report through microbiome and metabolome analysis that oral MOLE supplementation improves semen quality by beneficially altering the diversity and composition of the rumen microbiota, increasing the PUFA content, and improving the antioxidant capacity as well as promoting the secretion of testosterone in male cashmere goats. This study provided a theoretical basis for the application of MOLE and MOLP in the goat industry.

## Conclusions

This study confirmed that MOLP and MOLE can improve the antioxidant capacity of cashmere goats. In addition, MOLE had positive effects on the reproductive performance of cashmere goats, and these positive effects were attributed to the unique chemical composition contained in MOLE. When supplemented with 40 mg/kg BW, the semen quality of cashmere goats improved. This promotion is achieved by favorably altering the rumen microbiota composition, increasing PUFAs content, and promoting testosterone secretion. Therefore, MOLE can be used as a dietary additive to improve the quality of cashmere goat semen and has practical implications for improving the reproductive performance of goats.

## Data availability statement

The datasets presented in this study can be found in online repositories. The names of the repository/repositories and accession number(s) can be found below: https://www.ncbi.nlm.nih.gov/, PRJNA876959.

## Ethics statement

The animal study was reviewed and approved by Inner Mongolia Academy of Agricultural and Animal Husbandry Sciences (No. 200610-012).

## Author contributions

JL, YG, and BL designed the study. JL, TWu, and TWa conducted the experiments and performed the statistical analysis of the experimental data. YM, YL, SZ, YG, and BL discussed the results and provided valuable suggestions and comments to improve the manuscript. Finally, the paper was written by JL and was modified by BL. All authors read and approved the final manuscript.

## Supplementary material

Supplementary Figure 1 Effects of MOLE and MOLP supplementation on the diversity of rumen microorganisms of cashmere goats. **(A)** Rarefaction curve. **(B)** Chao1 index. **(C)** Shannon index. **(D)** Venn plot. **(E)** Microbial clustering based on the Binary-Jaccard distance metric, visualized by principal coordinate analysis.

Supplementary Figure 2 Pathway enrichment analysis performed using the significantly different rumen metabolites between MOLP and CON groups.

Supplementary Table 1 Ingredient and nutrient levels of the diets fed to male cashmere goats.

Supplementary Table 2 The nutrient composition per 100 g of *Moringa oleifera* leaf powder (MOLP).

Supplementary Table 3 Sample sequencing data statistics.

Supplementary Table 4 1006 Metabolites information overview table.

Supplementary Table 5 Differential rumen metabolites between MOLE and CON groups.

Supplementary Table 6 Differential rumen metabolites between MOLP and CON groups.
