# Supplementary material for: Moringa oleifera leaf ethanolic extract benefits cashmere goat semen quality via improving rumen microbiota and metabolome
Source: Front Vet Sci. 2023 Jan 27;10:1049093. doi: 10.3389/fvets.2023.1049093 (PMC9911920; doi:10.3389/fvets.2023.1049093)
Supplement: Supplementary Figure 1 — Effects of MOLE and MOLP supplementation on the diversity of rumen microorganisms of cashmere goats. (A) Rarefaction curve. (B) Chao1 index. (C) Shannon index. (D) Venn plot. (E) Microbial clustering based on the Binary-Jaccard distance metric, visualized by principal coordinate analysis. [file Data_Sheet_1.docx]

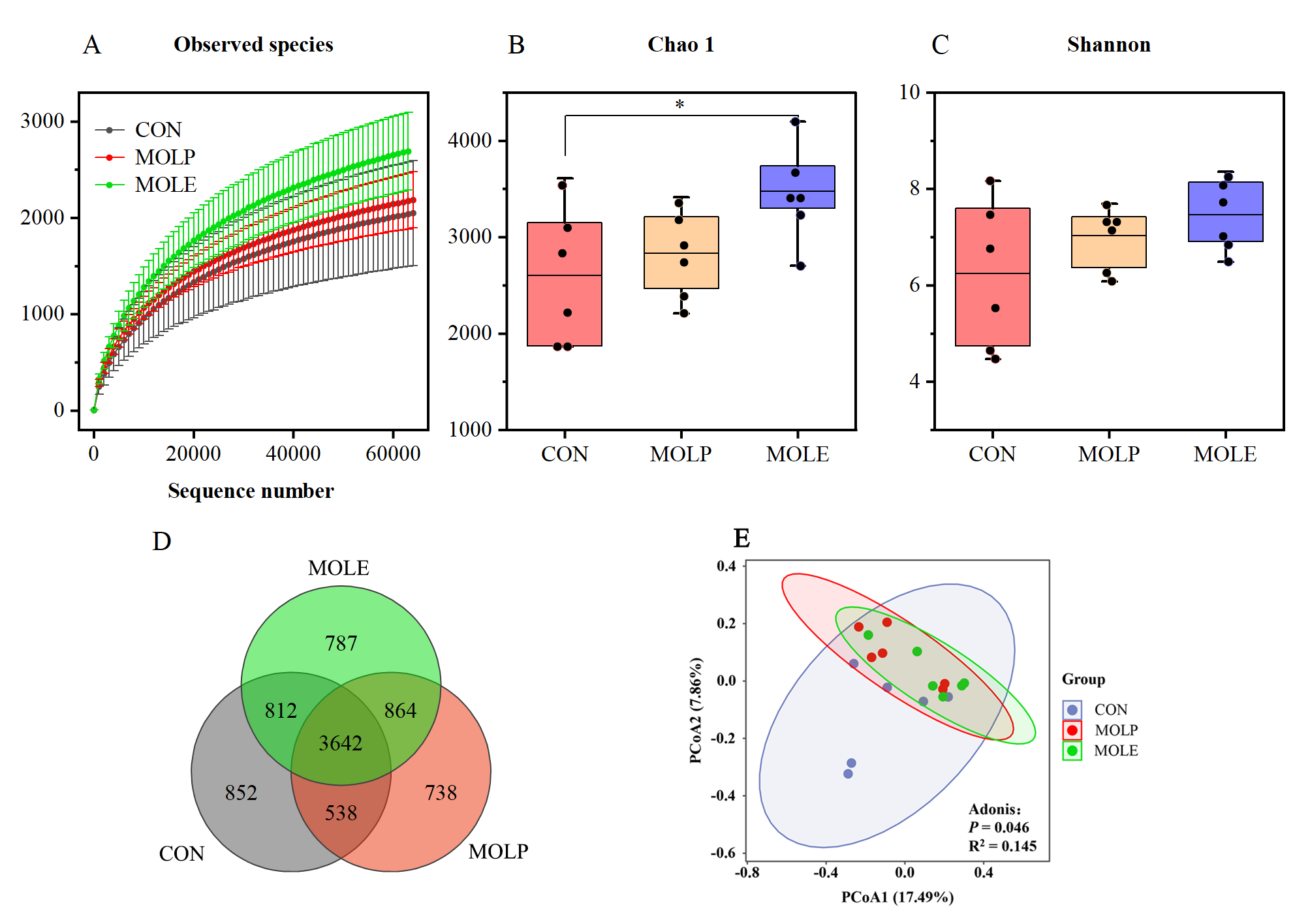


**Supplementary Figure 1** Effects of MOLE and MOLP supplementation on the diversity of rumen microorganisms of cashmere goats. (A) Rarefaction curve. (B) Chao1 index; Variation in diversity within the three groups. Sample diversity was measured at the OTU level (> 97% identity). The microbial community in the MOLE group displayed a significantly higher diversity than that displayed in the control group (Fisher's least significant difference test; **P* < 0.05), and somewhat higher than that displayed in the MOLP group, although not significant. (C) Shannon index. (D) Venn plot. (E) Microbial clustering based on the Binary-Jaccard distance metric, visualized by principal coordinate analysis. (Adonis R^2^ = 0.145, *P* = 0.046). The values in the boxplot are individual sample relative abundance from maximum to minimum.
