# Supplementary material for: Moringa oleifera leaf ethanolic extract benefits cashmere goat semen quality via improving rumen microbiota and metabolome
Source: Front Vet Sci. 2023 Jan 27;10:1049093. doi: 10.3389/fvets.2023.1049093 (PMC9911920; doi:10.3389/fvets.2023.1049093)
Supplement: Supplementary Figure 2 — Pathway enrichment analysis performed using the significantly different rumen metabolites between MOLP and CON groups. [file Data_Sheet_2.docx]

**Supplementary Figure 2** Pathway enrichment analysis performed using the significantly different rumen metabolites between MOLP and CON groups.
