# Supplementary material for: Moringa oleifera leaf ethanolic extract benefits cashmere goat semen quality via improving rumen microbiota and metabolome
Source: Front Vet Sci. 2023 Jan 27;10:1049093. doi: 10.3389/fvets.2023.1049093 (PMC9911920; doi:10.3389/fvets.2023.1049093)
Supplement: Supplementary Table 1 — Ingredient and nutrient levels of the diets fed to male cashmere goats. [file Table_1.DOCX]

**Supplementary Table 1** Ingredient and nutrient levels of the diets fed to male cashmere goats

| Items | Content |
| --- | --- |
| Ingredient (% dry matter basis) | |
| Alfalfa hay particles | 10 |
| Corn stalk | 20 |
| Sunflower seed hull | 10 |
| Corn | 24 |
| Wheat bran | 8.5 |
| Soybean meal | 8.3 |
| Distillers dried grains with solubles | 16 |
| Premix^a^ | 1 |
| CaHPO4 | 0.7 |
| NaCl | 0.5 |
| Limestone | 1 |
| Nutrition levels | |
| Digestible energy^b^, MJ/kg DM | 11.37 |
| Crude protein, g/kg DM | 154.3 |
| NDF, g/kg DM | 412.1 |
| ADF, g/kg DM | 214.3 |
| Calcium, g/kg DM | 9.46 |
| Phosphorus, g/kg DM | 4.71 |

^a^ Premix provides the following per kg of the diet: iron (Fe) 50 mg, copper (Cu) 27 mg, zinc (Zn) 50 mg, manganese (Mn) 30 mg, iodine (I) 1 mg, selenium (Se) 0.5 mg, cobalt (Co) 0.33 mg, vitamin A (VA) 6,000 IU, vitamin E (VE) 125 IU.

^b^ Digestible energy is calculated based on the ingredients of the diet and their digestible energy content, not based on the actual dry matter intake.
