# Supplementary material for: Moringa oleifera leaf ethanolic extract benefits cashmere goat semen quality via improving rumen microbiota and metabolome
Source: Front Vet Sci. 2023 Jan 27;10:1049093. doi: 10.3389/fvets.2023.1049093 (PMC9911920; doi:10.3389/fvets.2023.1049093)
Supplement: Supplementary Table 2 — The nutrient composition per 100 g of Moringa oleifera leaf powder (MOLP). [file Table_2.DOCX]

**Supplementary Table 2** The nutrient composition per 100g of *Moringa oleifera* leaf powder (MOLP)

| Amount Per 100g | Content |
| --- | --- |
| Water, % | 7.26 |
| Protein, g | 17.30 |
| Lipids, g | 4.90 |
| Fibre, g | 20.20 |
| Calcium, mg | 2233.07 |
| Phosphorus, mg | 210.53 |
