# Supplementary material for: Moringa oleifera leaf ethanolic extract benefits cashmere goat semen quality via improving rumen microbiota and metabolome
Source: Front Vet Sci. 2023 Jan 27;10:1049093. doi: 10.3389/fvets.2023.1049093 (PMC9911920; doi:10.3389/fvets.2023.1049093)
Supplement: Supplementary Table 3 — Sample sequencing data statistics. [file Table_3.DOCX]

**Supplementary Table 3** Sample sequencing data statistics

| Taxonomy | Sample ID | Clean Tags | Valid Tags | valid  Mean Length | valid  Percent |
| --- | --- | --- | --- | --- | --- |
| CON | 1 | 74186 | 69919 | 421 | 94.25% |
|  | 2 | 72740 | 68109 | 420 | 93.63% |
|  | 3 | 72630 | 68260 | 420 | 93.98% |
|  | 4 | 75158 | 70006 | 420 | 93.15% |
|  | 5 | 73111 | 65945 | 419 | 90.20% |
|  | 6 | 73448 | 64978 | 420 | 88.47% |
| MOLP | 1 | 73218 | 67682 | 420 | 92.44% |
|  | 2 | 74362 | 66988 | 419 | 90.08% |
|  | 3 | 72290 | 64800 | 418 | 89.64% |
|  | 4 | 74060 | 66237 | 420 | 89.44% |
|  | 5 | 73634 | 65320 | 420 | 88.71% |
|  | 6 | 71810 | 65252 | 415 | 90.87% |
| MOLE | 1 | 74282 | 68207 | 417 | 91.82% |
|  | 2 | 74193 | 65497 | 420 | 88.28% |
|  | 3 | 72088 | 63941 | 420 | 88.70% |
|  | 4 | 72920 | 65485 | 420 | 89.80% |
|  | 5 | 72084 | 63500 | 420 | 88.09% |
|  | 6 | 73783 | 65820 | 419 | 89.21% |
